# Role of sentinel lymph node biopsy in patients with early breast cancer undergoing mastectomy

**DOI:** 10.1093/bjsopen/zrag105

**Published:** 2026-09-04

**Authors:** Martin Heidinger, Giacomo Montagna, Florian S Halbeisen, Anna-Lena Eberhardt, Nadia Maggi, Marie Louise Frevert, Karol Dollt, Rama Kiblawi, Julie M Loesch, Jelena Winkler, Fabienne D Schwab, Christian Kurzeder, Tibor A Zwimpfer, Walter P Weber

**Affiliations:** Breast Clinic, University Hospital Basel, Basel, Switzerland; Gynaecology & Obstetrics, University Hospital Basel, Basel, Switzerland; Medical Faculty, University of Basel, Basel, Switzerland; Breast Service, Department of Surgery, Memorial Sloan Kettering Cancer Center, New York, NY, USA; Surgical Outcome Research Center, University Hospital Basel and University of Basel, Basel, Switzerland; Radiation Oncology, University Hospital Basel, Basel, Switzerland; Breast Clinic, University Hospital Basel, Basel, Switzerland; Medical Faculty, University of Basel, Basel, Switzerland; Department of Medical Epidemiology and Biostatistics, Karolinska Institutet, Stockholm, Sweden; Breast Clinic, University Hospital Basel, Basel, Switzerland; Gynaecology & Obstetrics, University Hospital Basel, Basel, Switzerland; Medical Faculty, University of Basel, Basel, Switzerland; Breast Clinic, University Hospital Basel, Basel, Switzerland; Breast Clinic, University Hospital Basel, Basel, Switzerland; Gynaecology & Obstetrics, University Hospital Basel, Basel, Switzerland; Medical Faculty, University of Basel, Basel, Switzerland; Breast Clinic, University Hospital Basel, Basel, Switzerland; Gynaecology & Obstetrics, University Hospital Basel, Basel, Switzerland; Radiation Oncology, University Hospital Basel, Basel, Switzerland; Breast Clinic, University Hospital Basel, Basel, Switzerland; Gynaecology & Obstetrics, University Hospital Basel, Basel, Switzerland; Medical Faculty, University of Basel, Basel, Switzerland; Breast Clinic, University Hospital Basel, Basel, Switzerland; Gynaecology & Obstetrics, University Hospital Basel, Basel, Switzerland; Medical Faculty, University of Basel, Basel, Switzerland; Gynaecology & Obstetrics, University Hospital Basel, Basel, Switzerland; Medical Faculty, University of Basel, Basel, Switzerland; Breast Clinic, University Hospital Basel, Basel, Switzerland; Medical Faculty, University of Basel, Basel, Switzerland

**Keywords:** breast-conserving surgery, CDK 4/6 inhibitors, ribociclib, abemaciclib, regional nodal irradiation

## Abstract

**Background:**

The oncological non-inferiority of sentinel lymph node biopsy omission in patients with small, clinically and imaging node-negative (cN0/iN0) breast cancer has been demonstrated for patients undergoing breast-conserving surgery and adjuvant radiotherapy, but not for those undergoing mastectomy. This single-centre, retrospective cohort study compared nodal involvement and adjuvant therapy indications in these patients by use of mastectomy *versus* breast-conserving surgery.

**Methods:**

Patients with cT1-T2, cN0/iN0, hormone receptor-positive/human epidermal growth factor receptor 2-negative early breast cancer who underwent upfront surgery including sentinel lymph node biopsy between January 2014 and December 2024 were included. Adjuvant therapy indications were estimated using monarchE (abemaciclib), NATALEE (ribociclib), TAILORx and RxPONDER (chemotherapy), and MA.20 and EORTC22922/10925 (nodal irradiation) eligibility criteria. Multivariable logistic regression models identified factors associated with nodal involvement and adjuvant treatment indications.

**Results:**

Among 454 patients, 145 (31.9%) underwent mastectomy who more frequently had multifocal tumours (23.5% *versus* 9.7%), lobular subtype (21.4% *versus* 11.3%), and grade 2–3 differentiation (73.8% *versus* 63.8%) compared with those who underwent breast-conserving surgery. Neither nodal involvement (18.6% *versus* 14.9%; *P* = 0.149) nor missed indications for adjuvant abemaciclib (7.6% *versus* 6.1%; *P* = 0.550), ribociclib (9.7% *versus* 7.8%; *P* = 0.586), and nodal irradiation (4.8% *versus* 4.5%; *P* = 1.000) differed between patients who underwent mastectomy or breast-conserving surgery. Lymphovascular invasion was independently associated with nodal involvement (odds ratio 6.8, 95% confidence interval 3.7 to 12.8; *P* < 0.001) and adjuvant treatment indications (CDK4/6 inhibitors: odds ratio 5.1, 2.6 to 10.1; *P* < 0.001; nodal irradiation: odds ratio 7.1, 3.6 to 14.4; *P* < 0.001), whereas type of surgery was not.

**Conclusions:**

In patients with cT1-T2, cN0/iN0, hormone receptor-positive/human epidermal growth factor receptor 2-negative breast cancer, type of breast surgery was not associated with nodal involvement and adjuvant therapy.

## Teaser

It remains unclear whether patients who undergo mastectomy but otherwise fulfil eligibility criteria for the omission of sentinel lymph node biopsy present with higher nodal involvement or a change in adjuvant therapy. Among 454 patients, neither the proportion of node-positivity (18.6% *versus* 14.9%) nor adjuvant therapy differed in patients who underwent a mastectomy *versus* breast-conserving surgery, respectively.

## Introduction

There is mounting evidence that the omission of sentinel lymph node biopsy (SLNB) in appropriately selected patients with breast cancer (BC) does not result in oncologically inferior outcomes. Oncological non-inferiority and improved quality of life upon SLNB omission was recently reported in three phase-III randomized controlled trials—SOUND, INSEMA, and BOOG 2013-08. Patients included presented with cT1/2 BC, and were found to be node negative both clinically and by means of axillary ultrasound (cN0/iN0)^1–6^. All patients in these trials underwent breast-conserving therapy (BCT; including breast-conserving surgery (BCS) and adjuvant radiotherapy). The majority had hormone receptor (HR)-positive and human epidermal growth factor receptor 2 (HER2)-negative BC.

Mastectomy remains a frequently performed procedure, with many women electing to undergo this procedure despite it being a more extensive surgery compared with BCS^7,8^. Oncologically, mastectomy provides similar local control compared with BCT^9,10^. However, tumour stage may generally be more advanced in patients undergoing a mastectomy, potentially impacting adjuvant treatment decisions^11–13^. Nevertheless, several reasons to opt for a mastectomy exist, and although these patients may not necessarily present with higher-risk tumours, they may be subjected to more extensive axillary surgery, as mastectomy was not allowed in most SLNB omission trials. The patient cohort included in the pivotal SLN omission trials represents a selected, low-risk population. Consequently, if nodal disease burden and adjuvant treatment may not be impacted by the type of surgery in this patient population, SLNB omission can be evaluated in patients undergoing mastectomy.

The present study addressed the question of whether nodal disease burden differed between patients with HR-positive/HER2-negative BC who underwent mastectomy *versus* BCS and were otherwise eligible for SLNB omission. Furthermore, the present study aims to compare potentially missed adjuvant treatment options in these patients by the type of surgery.

## Methods

### Study design

Consecutive patients with ≤ cT2, node-negative (cN0/iN0), HR-positive/HER2-negative BC who underwent upfront BCS or mastectomy and SLNB at the University Hospital of Basel, Switzerland, between January 2014 and December 2024 were included. Data for this analysis was extracted on 2 June 2025.

Participants were prospectively identified during preoperative consultations at the University Hospital Basel and were entered into the institutional database upon providing written informed consent. The database is integrated in the online Good Clinical Practice compliant data management system secuTrial©. The study is reported according to the STROBE guidelines^14^.

### Eligibility for adjuvant treatment

Eligibility for adjuvant treatments is summarized in *Table 1*. Candidacy for adjuvant abemaciclib was assessed according to eligibility criteria of the monarchE trial^15^. Candidacy for adjuvant ribociclib was assessed according to eligibility criteria of the NATALEE trial. For the present study, patients eligible for treatment with ribociclib were identified based on nodal criteria^23,24^. Criteria for the indication for adjuvant chemotherapy in pre- and postmenopausal women were evaluated according to the TAILORx and RxPonder trials, and according to institutional guidelines^17,18^. Finally, indications for regional nodal irradiation were evaluated according to MA20 and EORTC22922/10925 trials, as well as according to international and institutional guidelines^19–22^. These indications were further categorized as absolute indications (strong recommendation) and relative indications (conditional recommendation).

**Table 1:** Adjuvant treatment indications

Treatment	Indication
Abemaciclib^15^ monarchE	≥ 4 positive lymph nodes 1–3 positive lymph nodes and either grade 3 disease, or tumour size ≥ 5 cm, or Ki67 ≥ 20%
Ribociclib^16^ NATALEE	pT1pN1 (excluding pN1mi) pT2pN1*
Chemotherapy TAILORx^17^, RxPONDER^18^, institutional guidelines	Premenopausal women ≥ 1 positive lymph node (excluding pN1mi) Oncotype DX recurrence score ≥ 16 Postmenopausal women ≥ 4 positive lymph nodes Oncotype DX recurrence score ≥ 26
Nodal irradiation MA.20^19^, EORTC22922/10925^20^, international guidelines^21, 22^, institutional guidelines	Absolute indication: Breast-conserving surgery† > 3 macrometastatic nodes 1–3 macrometastatic nodes and medially/centrally located tumour 1–3 macrometastatic nodes and ≥ pT2 and grade 3 Mastectomy† ≥ 2 macrometastatic nodes Relative indication: Breast conserving surgery† ≥ 1 macrometastatic node Mastectomy† ≥ 1 macrometastatic node

^*^Provided that no additional criteria would render the pT2 status *per se* eligible for ribociclib treatment (that is, grade 3; or grade 2 plus Ki67 ≥ 20% or grade 2 plus Oncotype DX recurrence score ≥ 26), irrespective of nodal status. †Provided that no non-nodal criteria would render the patient eligible for nodal radiotherapy (premenopausal and medially/centrally located tumour and grade 3 and ≥ pT2; or premenopausal and medially/centrally located tumour and ≥ pT3).

### Covariates

The clinical tumour stage was evaluated by means of routine clinical examination and imaging including mammography and ultrasound. All patients underwent axillary ultrasound for nodal staging as institutional standard over the entire study period. Tumour stage was reported according to the American Joint Committee on Cancer Cancer Staging Manual 8th Edition^25^. The performance of Oncotype DX recurrence score testing was at the discretion of the treating physician. During the study period no other genomic tests were used.

### Aims

The primary aim of this study was to assess whether nodal disease burden differs between patients who underwent BCS or mastectomy and otherwise met the eligibility criteria for SLNB omission. A secondary aim was to assess the potential impact of SLNB omission on adjuvant treatment indications in these patients.

### Statistical analysis

Patient, tumour, and treatment characteristics were summarized using descriptive statistics. The results are presented as median (interquartile range (i.q.r.)) for continuous variables and compared using the Wilcoxon rank-sum test. Categorical variables are presented as absolute frequencies with percentages and compared between groups via Fisher’s exact test. Multivariable logistic regression models including clinicopathological variables were used to identify factors associated with node positivity, CDK4/6 inhibitor eligibility, and nodal irradiation. All statistical analyses were performed using R (version 4.3.2).

### Ethical approval

This research project complies with the guidelines for human studies according to the Declaration of Helsinki as revised in 2013. The study protocol was approved by the Ethics Committee of Northwest and Central Switzerland (Ethikkommission Nordwest und Zentralschweiz, EKNZ; Approval number 2016-01525), and all subjects gave their written informed consent.

## Results

### Patient and tumour characteristics

**Figure 1:**
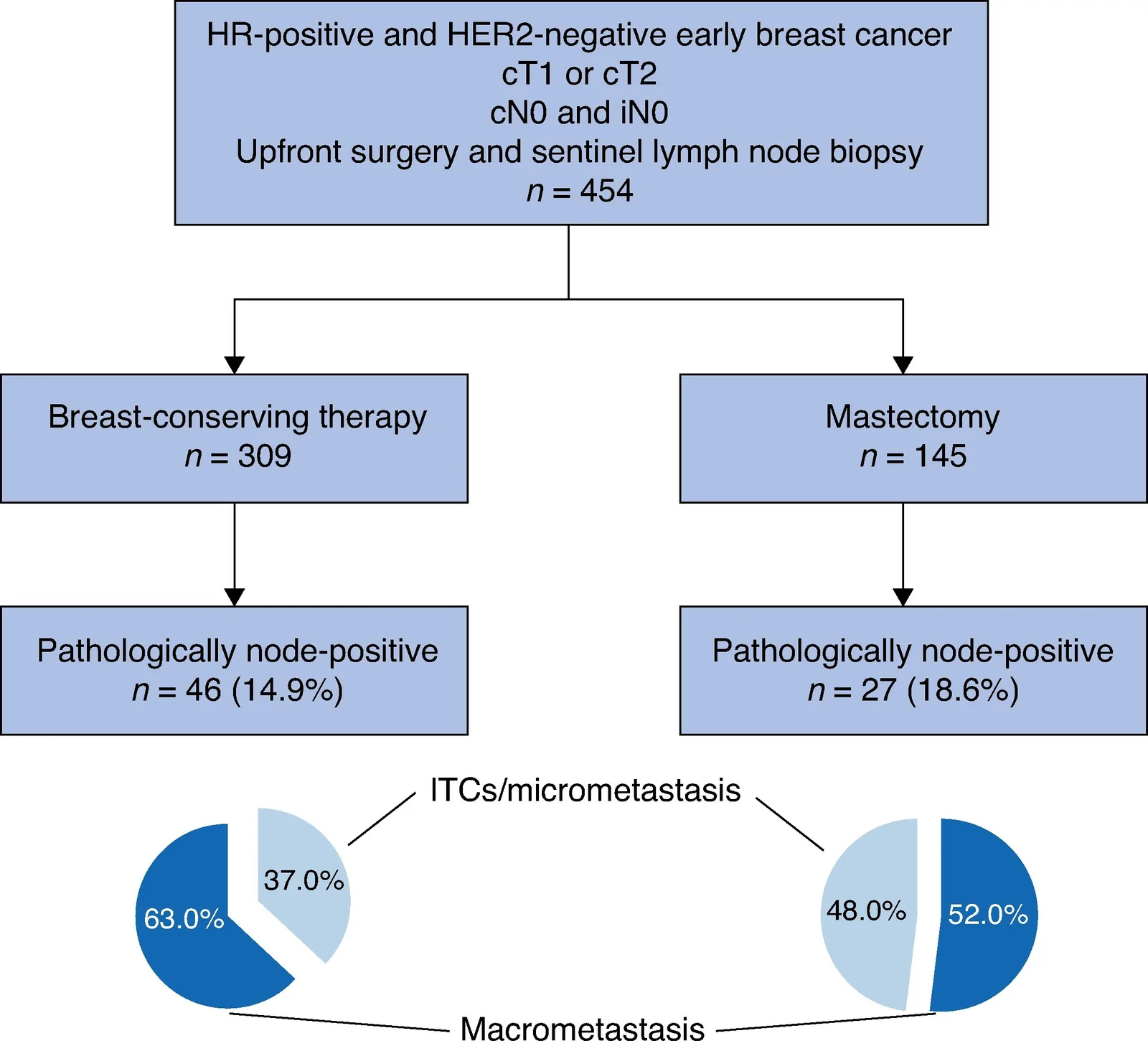
Study flow

A total of 454 patients met the eligibility criteria, of whom 309 (68.1%) underwent BCS and 145 (31.9%) mastectomy (*Fig. 1*). Patients who underwent a mastectomy tended to have larger tumours (cT2: 37.2% *versus* 27.8%; *P* = 0.050), which were more frequently multifocal (23.5% *versus* 9.7%; *P* < 0.001), of lobular histotype (21.4% *versus* 11.3%; *P* = 0.008), and with a higher tumour grade (grade II and III: 73.8% *versus* 63.8%; *P* = 0.022; *Table 2*).

**Table 2:** Patient, tumour, and treatment characteristics

Characteristic	Breast-conserving surgery *n* = 309	Mastectomy *n* = 145	P*
Age at surgery (years), median (i.q.r.)	61 (53–70)	60 (49–74)	0.635
**Ethnicity**			
White/Caucasian	282 (91.3%)	136 (93.8%)	0.457
Other	27 (8.7%)	9 (6.2%)	
**Menopausal status**			
Premenopausal	66 (21.4%)	45 (31.0%)	0.021
Postmenopausal	241 (78.0%)	97 (66.9%)	
Missing	2 (0.7%)	3 (2.1%)	
**Clinical tumour stage**			
cT1	223 (72.2%)	91 (62.8%)	0.050
cT2	86 (27.8%)	54 (37.2%)	
**Focality**			
Unifocal	279 (90.3%)	111 (76.6%)	< 0.001
Multifocal	30 (9.7%)	34 (23.4%)	
**Histological type**			
Invasive lobular	34 (11.0%)	32 (22.1%)	0.006
Invasive ductal	232 (75.1%)	101 (69.7%)	
Other	43 (13.9%)	12 (8.3%)	
**Ki67**			
Median (i.q.r.)	10 (5–20)	15 (10–20)	0.036
< 20%	214 (69.3%)	90 (62.1%)	0.248
≥ 20%	92 (29.8%)	54 (37.2%)	
Missing	3 (1.0%)	1 (0.7%)	
**Tumour grade**			
I	112 (36.2%)	38 (26.2%)	0.026
II	160 (51.8%)	78 (53.8%)	
III	37 (12.0%)	29 (20.0%)	
**Lymphovascular invasion**			
L0	252 (81.6%)	114 (78.6%)	0.485
L1	55 (17.8%)	31 (21.4%)	
Missing	2 (0.6%)	0	
**Pathologic tumour stage**			
pT0/pTis	2 (0.7%)	1 (0.7%)	0.189
pT1	218 (70.6%)	95 (65.5%)	
pT2	84 (27.2%)	43 (29.7%)	
pT3	5 (1.6%)	6 (4.1%)	
**Pathologic nodal stage**			
pN0	263 (85.1%)	118 (81.4%)	0.162
pN+	46 (14.9%)	27 (18.6%)	
**Type of initial breast surgery**			
Breast-conserving surgery	309 (100%)	24 (16.6%)	< 0.001
Mastectomy	0	121 (83.4%)	
**Type of final axillary surgery**			
SLNB	299 (96.8%)	140 (96.6%)	0.836
Axillary dissection	10 (3.2%)	5 (3.4%)	
**Sentinel lymph node localization**			
Single tracer	210 (68.0%)	108 (74.5%)	0.187
Dual tracer	99 (32.0%)	37 (25.5%)	
**Number of sentinel lymph nodes removed**			
Median (i.q.r.)	2 (1–3)	2 (2–3)	0.019
0	0	1 (0.7%)	0.004
1	115 (37.2%)	33 (22.8%)	
2	96 (31.1%)	59 (40.7%)	
≥ 3	98 (31.7%)	52 (35.9%)	
**Number of positive lymph nodes**			
Median	0	0	0.283
0	263 (85.1%)	118 (81.4%)	0.149
1	37 (12.0%)	19 (13.1%)	
2	6 (1.9%)	7 (4.8%)	
3	3 (1.0%)	0	
≥ 4	0	1 (0.7%)	
**Oncotype DX**			
Performed	83 (26.9%)	42 (29.0%)	0.653
Recurrence score ≥ 26, *n* of *N* (%)	23 of 83 (27.7%)	7 of 42 (16.7%)	0.266

Values are *n* (%) unless otherwise stated. i.q.r., interquartile range; SLNB, sentinel lymph node biopsy. *Continuous variables were compared using Wilcoxon rank-sum test; categorical variables were compared using Fisher’s exact test.

### Nodal disease burden

The type of final axillary surgery did not differ between the groups, with 96.8% of patients after BCS and 96.6% of patients who underwent mastectomy having undergone SLNB, respectively. The median number of removed lymph nodes was higher in patients who underwent mastectomy (BCS: 2 (i.q.r. 1–3); mastectomy: 2 (i.q.r. 2–3); *P* = 0.013); however, the proportion of patients with positive lymph nodes was comparable between groups (BCS 14.9%; mastectomy 18.6%; *P* = 0.149). Macrometastatic disease was present in 63.0% of patients who underwent BCS and in 52.0% of patients who underwent mastectomy (*P* = 0.451).

**Figure 2:**
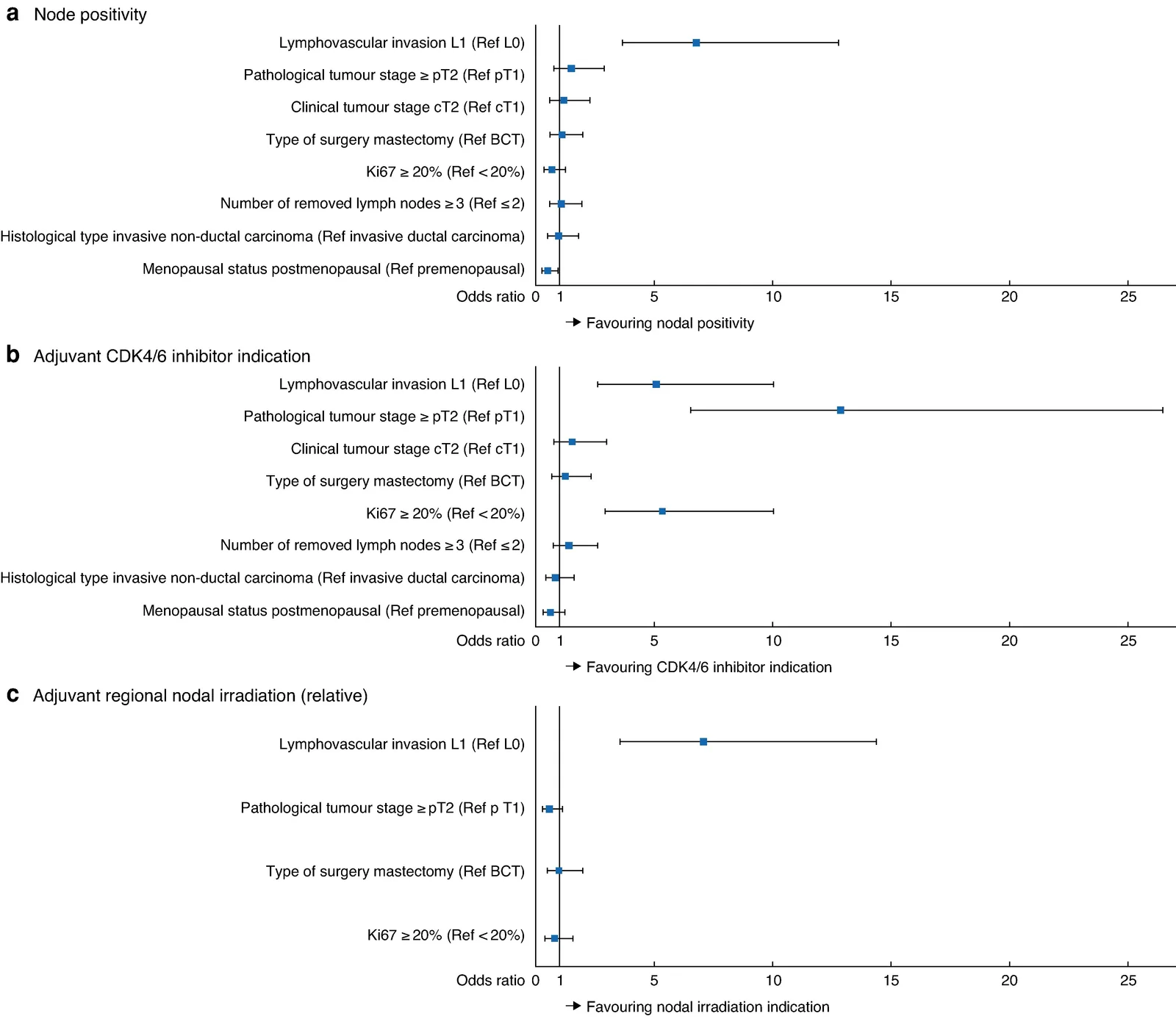
Multivariable logistic regression analysis of predictors for node positivity, adjuvant CDK4/6 inhibitor indication, and adjuvant regional nodal irradiation (relative indication) **a** node positivity, **b** adjuvant CDK4/6 inhibitor indication, and **c** adjuvant regional nodal irradiation (relative indication). CDK4/6, cyclin-dependent kinase 4/6; Ref, reference; BCT, breast-conserving therapy.

A higher likelihood of node-positive disease was observed in case of lymphovascular invasion (odds ratio (OR) 6.8, 95% confidence interval (c.i.) 3.7 to 12.8; *P* < 0.001), whereas postmenopausal status had a lower likelihood (OR 0.5, 0.3 to 1.0; *P* = 0.041, *Fig. 2a*). The type of breast surgery was not independently associated with node positivity (reference BCS, OR 1.1, 0.6 to 2.0; *P* = 0.708).

### CDK4/6 inhibitor eligibility based on nodal criteria

Eligibility for abemaciclib was present in 6.6% of patients, and eligibility for ribociclib, based on nodal criteria, was present in 8.4%. No difference was observed for CDK4/6 inhibitor indication based on nodal criteria by type of breast surgery (abemaciclib: BCS 6.1%, mastectomy 7.6%; *P* = 0.550; ribociclib: BCS 9.7%, mastectomy 7.8%; *P* = 0.586, *Table 3*). The number needed to undergo a SLNB to identify one meeting the criteria for abemaciclib was 17 (95% c.i. 11 to 27) after BCS and 14 (8 to 26) after a mastectomy; it was 13 (9 to 20) and 11 (7 to 19) for ribociclib based on nodal criteria, respectively. These results were consistent in the subgroup of patients with cT1 BC.

**Table 3:** Adjuvant treatment indications based on nodal criteria

	Breast-conserving surgery	Mastectomy	P*
**CDK4/6 inhibitor**			
Abemaciclib	19 of 309 (6.1%)	11 of 145 (7.6%)	0.550
Abemaciclib, cT1	10 of 223 (4.5%)	4 of 91 (4.4%)	1.000
Ribociclib	24 of 309 (7.8%)	14 of 145 (9.7%)	0.586
Ribociclib, cT1	18 of 223 (8.1%)	9 of 91 (9.9%)	0.658
**Nodal irradiation**			
Absolute indication			
All patients	14 of 309 (4.5%)	7 of 145 (4.8%)	1.000
cT1	8 of 223 (3.6%)	6 of 91 (6.6%)	0.242
Relative indication			
All patients	29 of 309 (9.4%)	15 of 145 (10.3%)	0.736
cT1	18 of 223 (8.1%)	9 of 91 (9.9%)	0.658

Values are *n* of *N* (%) unless otherwise stated. CDK4/6, cyclin-dependent kinase 4/6. *Fisher’s exact test.

Factors independently associated with CDK4/6i eligibility were found to be ≥ pT2 (OR 12.9, 95% c.i. 6.5 to 26.5; *P* < 0.001), Ki67 ≥ 20% (OR 5.3, 2.9 to 10.0; *P* < 0.001), and lymphovascular invasion (OR 5.1, 2.6 to 10.1; *P* < 0.001). Mastectomy was not associated with CDK4/6i eligibility (OR 1.3, 0.7 to 2.3; *P* = 0.481, *Fig. 2b*).

### Indication for adjuvant chemotherapy based on nodal criteria

In the postmenopausal setting, no patient in the BCS group had high nodal burden, whereas 1 of 97 patients (1.0%) who underwent mastectomy presented with pN2 disease and may have missed an adjuvant chemotherapy indication without SLNB.

In the premenopausal setting, 3 of 66 patients (4.5%) in the BCS group who presented with a recurrence score < 16 and 1 of 45 patients (2.2%) in the mastectomy group may have missed a chemotherapy indication if no SLNB had been performed.

### Indications for nodal irradiation based on nodal criteria

Absolute indications (*Table 1*) for nodal irradiation based on nodal criteria were present in 4.5% of patients after BCS and 4.8% after mastectomy (*P* = 1.000). Relative indications were present in 9.4% and 10.3% (*P* = 0.736), respectively. Results were consistent in patients with cT1 BC.

Lymphovascular invasion (OR 7.1, 95% c.i. 3.6 to 14.4; *P* < 0.001) was identified as an independent factor predicting nodal irradiation indication. The type of breast surgery was not associated with nodal irradiation (reference BCS, OR 1.0, 0.5 to 1.9; *P* = 0.996, *Fig. 2c*).

## Discussion

Patients undergoing mastectomy were mostly not included in contemporary axillary surgery de-escalation trials. Whereas the ACOSOG-Z0011 study^26^ only included patients who underwent BCT, the AMAROS trial^27^ included 17.4% of patients with mastectomy and the SENOMAC trial^28^ included 36.2%, which reflects current real-world mastectomy rates^7,8^. The SOUND^1^, INSEMA^2^, and BOOG 2013-08^3^ trials are the first in a series of ongoing trials to report the oncological non-inferiority of SLNB omission in selected patients with low-risk BC. However, patients undergoing mastectomy were excluded from all three of these trials and most other ongoing studies enrolling similar patient populations^29,30^. One exemption is the VENUS trial (NCT05315154), which includes patients with early BC up to 5 cm, cN/iN0 in the upfront surgery or post-neoadjuvant setting who undergo BCS or mastectomy. Recruitment is ongoing and results are not expected before 2029^31^.

The rationale for the exclusion of patients undergoing mastectomy is based on the technical challenge to return to the operating room to perform SLNB in a second procedure in case of final pathology-based exclusion criteria for SLNB omission, such as larger than expected primary tumours or higher grade. Furthermore, patients are commonly selected for mastectomy based on higher risk features, such as larger or multicentric tumours, and the potential lack of any adjuvant radiotherapy with accidental treatment of the axilla. However, multiple reasons to opt for a mastectomy exist, including an unfavourable breast-to-tumour ratio, pathogenic germline BRCA variants, contraindications to radiotherapy, radiotherapy-associated treatment burden, fear of potential radiotherapy side-effects, or the patient’s preference^13,32–34^. Therefore, many patients undergoing mastectomy who otherwise meet the eligibility criteria for SLNB omission may be surgically overtreated. As shown in the AMAROS^35^ and SENOMAC^28^ trials, the subgroup of patients who underwent mastectomy equally experienced no oncological demise compared with patients who received BCT.

Therefore, the aim of the present study was to assess the potential impact of mastectomy on nodal disease burden and adjuvant treatment in patients who otherwise meet SLNB omission eligibility. In the present cohort, neither the proportion of patients with node-positive BC nor the proportion with macrometastatic disease differed between patients who underwent BCS and those treated by mastectomy. Furthermore, no differences were observed in the proportions of potentially missed adjuvant treatments, including CDK4/6i, chemotherapy, and nodal irradiation.

In the present study, although more lymph nodes were removed in the cohort of patients who underwent mastectomy, the proportion of node positivity and the number and size of involved lymph nodes did not differ between those who underwent BCS and those who underwent mastectomy. This is consistent with the findings of Chen *et al*.^36^, who reported node positivity in 7.6% and 8.2% following BCS or mastectomy, respectively, for cT1, cN0, HR-positive/HER2-negative BC. Therefore, in selected cases, the predictive value of axillary clinical examination and ultrasound do not appear to differ between patients planned to undergo BCS or mastectomy. The sole factor associated with lymph node involvement in the present study was lymphovascular invasion, which has been shown to predict nodal metastasis^37,38^.

Aside from differences in nodal involvement, the potential impact of SLNB omission on adjuvant treatment in patients undergoing BCS or mastectomy was investigated. Adjuvant CDK4/6 inhibitors, as well as adjuvant chemotherapy, were shown to provide oncological benefits, irrespective of the type of surgery^15,16^. Recommendations for nodal irradiation differ between patients who undergo BCS and mastectomy^19–22^. Nodal information forms part of the eligibility criteria for all three treatment modalities (*Table 1*). Importantly, as the aim was to assess the rate of missed indications for any of the treatments investigated, patients with node-positive disease who would have received a treatment recommendation based on the primary tumour characteristics anyway were excluded. None of the treatments investigated was found to be missed more frequently if SLNB would be omitted in the total cohort of patients who underwent a mastectomy, as well as in the subgroup of patients with cT1 BC. Whether to treat individuals with small, low-risk BC with adjuvant CDK4/6 inhibitors is currently a matter of debate^39,40^. However, CDK4/6 inhibitor benefit was demonstrated across all subgroups, including patients with stage II disease^15,16,24,39,40^. All three of the reported trials^1–3^ on the oncological safety of SLNB omission had finished recruitment by the time CDK4/6 inhibitors were approved in the adjuvant setting. Therefore, no information on differences in the use of these treatments and their outcomes is available from prospective trials. A recent retrospective cohort study demonstrated that, for patients meeting SOUND/INSEMA eligibility criteria, the potential increase in invasive disease-free survival events upon SLNB omission and missed treatment with adjuvant CDK4/6i was 0.2–0.3%, based on the results of the 5-year follow-up data for abemaciclib and ribociclib in patients with stage II BC^41^. Therefore, a similar minimal impact on oncological outcomes may be assumed for patients who undergo mastectomy but otherwise fulfil the eligibility criteria for SLNB omission. In all three randomized clinical trials (RCTs) on SLNB omission, numerically more patients received adjuvant chemotherapy after SLNB^1–3^. There were generally few missed chemotherapy indications if no SLNB had been performed in the present study, without obvious difference between patients who underwent BCS or mastectomy.

Finally, the absolute and relative indications for nodal irradiation were analysed. To date, no overall survival benefit was observed for nodal irradiation in any of the three RCTs, namely MA.20^19^, EORTC 22922/10925^20^, and SUPREMO^42^. This explains the significant heterogeneity in clinical practice. Therefore, eligibility for nodal irradiation was not solely assessed based on trial eligibility criteria but incorporated international and institutional guidelines. The analysis was structured according to a framework distinguishing absolute and relative indications. The proportion of missed absolute indications was below 5%, and around 10% for relative indications, irrespective of the type of breast surgery. Of the reported SLNB omission trials, only SOUND allowed for partial breast irradiation in the form of ELIOT intraoperative radiotherapy, whereas INSEMA and BOOG 2013-08 required whole-breast radiotherapy^1–3^. In the INSEMA trial^43^, 50% of patients received a potentially therapeutic radiotherapy dose to level I of the axilla, whereas the proportion of patients receiving incidental axillary radiotherapy was low in the BOOG 2013-08 trial^44^, yielding similarly excellent oncological results. Therefore, the impact of nodal irradiation on oncological control after SLNB omission can be considered low.

As for node positivity, lymphovascular invasion was found to be associated with indications for adjuvant treatment. In clinical practice, this poses the difficulty that lymphovascular invasion detection in preoperative biopsy specimens has a high failure rate and is therefore not routinely evaluated in many settings^45^. However, the detection of lymphovascular invasion could prompt the recommendation to undergo SLNB, as results of the present study suggest that lymphovascular invasion is independently associated with higher nodal involvement and missed adjuvant treatment in the case of SLNB omission. Means of improving diagnosis include standardized tissue handling and examination, immunohistochemical staining, imaging-omics and nomograms, and predictive models using artificial intelligence^46–48^. Considering the results of the present study, enhanced prediction of lymphovascular invasion could help to stratify patients further as candidates for SLNB omission, regardless of the type of breast surgery performed. Alternative approaches may include technical solutions enabling delayed SLNB, such as superparamagnetic iron oxide, which can mark lymph nodes for up to 30 days and has been validated in patients with ductal carcinoma *in situ*^49^.

Current guidelines recommend SLNB omission in the patient population that was represented in the pivotal trials. These include women 50 years of age or older, with cT1, cN0/iN0, grade 1–2, HR-positive/HER2-negative BC^50,51^. Based on the present study, if these patients were to undergo mastectomy, they may represent a potential target group for extending SLNB omission eligibility.

This study has some limitations. Although SLNB omission trials included patients with cT2 tumours, the BOOG 2013-08 trial had the highest inclusion rate, representing a minority of 17%. Nevertheless, these patients were included in the present study, as both patients and treating physicians may base treatment decisions on the eligibility criteria of these trials. To accommodate for the cohort of patients included in the SLNB omission trials, subgroup analyses of patients with cT1 tumours were performed. These analyses showed consistent results compared with the total cohort.

Furthermore, the decisions to undergo mastectomy could not be further stratified, for example, according to patient preference *versus* clinical indications. Although this could potentially hinder the further selection of lower-risk patients who underwent a mastectomy, it represents real-world clinical practice.

Finally, as a retrospective single-centre analysis, this study is subject to certain limitations regarding generalizability, potential selection bias, and statistical power to detect small differences between the groups.

In patients with cT1-T2, cN0/iN0, HR-positive/HER2-negative BC, undergoing mastectomy was not associated with a higher proportion of node positivity. Moreover, the likelihood of potentially missed adjuvant treatment indications upon SLNB omission did not differ in patients who underwent a BCS compared with those treated by mastectomy. These findings support the consideration of SLNB omission in appropriately selected patients undergoing mastectomy, particularly postmenopausal women with low-risk tumours. Further investigation into extending SLNB omission eligibility to patients undergoing mastectomy is warranted.

## Data Availability

The data that support the findings of this study are available from the corresponding author upon reasonable request.
